# Narrowing of the Audiovisual Temporal Binding Window Due To Perceptual Training Is Specific to High Visual Intensity Stimuli

**DOI:** 10.1177/2041669520978670

**Published:** 2021-02-17

**Authors:** Ryan P. Horsfall, Sophie M. Wuerger, Georg F. Meyer

**Affiliations:** Department of Psychology, 4591 University of Liverpool, United Kingdom; Division of Neuroscience & Experimental Psychology, University of Manchester, United Kingdom; Department of Psychology, 4591 University of Liverpool, United Kingdom

**Keywords:** temporal binding window, multisensory, audiovisual, perceptual learning

## Abstract

The temporal binding window (TBW), which reflects the range of temporal offsets in which audiovisual stimuli are combined to form a singular percept, can be reduced through training. Our research aimed to investigate whether training-induced reductions in TBW size transfer across stimulus intensities. A total of 32 observers performed simultaneity judgements at two visual intensities with a fixed auditory intensity, before and after receiving audiovisual TBW training at just one of these two intensities. We show that training individuals with a high visual intensity reduces the size of the TBW for *bright* stimuli, but this improvement did not transfer to dim stimuli. The reduction in TBW can be explained by shifts in decision criteria. Those trained with the dim visual stimuli, however, showed no reduction in TBW. Our main finding is that perceptual improvements following training are specific for high-intensity stimuli, potentially highlighting limitations of proposed TBW training procedures.

Perceptual learning refers to improvements in the processing of sensory information brought on through experience. Behavioural performance improvements (Fahle, 1997; Herzog & Fahle, 1997) are typically complemented by structural and functional changes in primary sensory cortices (for a review, see Fahle & Poggio, 2002). However, perceptual learning has been shown to affect both perceptual sensitivity (McGovern et al., 2012) and decision criteria (Bang & Rahnev, 2017; Herzog et al., 2006).

Recent research on the integration of auditory and visual stimuli suggests that multisensorial binding mechanisms are plastic and can be modulated by rapid (Noel et al., 2016; Simon et al., 2017; Van der Burg et al., 2013) and sustained exposure (Di Luca et al., 2009; Fujisaki et al., 2004; Vroomen et al., 2004). Temporal proximity between the auditory and visual stimuli dictates whether these stimuli are integrated into a unified percept, that is, the closer in time the stimuli are, the more likely it is that they are combined. This temporal binding window (TBW) within which audiovisual stimuli are perceived as simultaneous is obtained with a simultaneity judgement (SJ) task where observers indicate whether auditory and visual stimuli with varying temporal offsets appear simultaneous.

From a functional perspective, it is desirable to discard low-level stimulus features such as stimulus intensity when judging whether an auditory and a visual event are simultaneous, as these stimulus attributes affect peripheral processing but do not reflect physical simultaneity. Light travels considerably faster than sound so that two simultaneously emitted stimuli will be detected by sensory cells with a distance-dependent time delay. Furthermore, the transduction between sensory cells and primary cortices also varies by modality, with considerably shorter latencies in the auditory than in the visual system (Raij et al., 2010). In addition to signal reception delays, there are differences in processing times as a function signal frequency in the auditory domain (Woods et al., 1993) or colour for visual signals (Pollack, 1968). A major determinant for processing delays is signal intensity (Lakhani et al., 2012; Nissen, 1977), evident in the intensity dependency of the point of subjective simultaneity (Leone & Mccourt, 2015), which reflects the stimulus offset asynchrony (SOA) at which audiovisual stimuli are most likely to be perceived as simultaneous.

The TBW absorbs some of the variability in arrival times brought about by peripheral processing differences (for a review, see Wallace & Stevenson, 2014). The TBW is plastic across the lifespan (Hillock-Dunn & Wallace, 2012; Noel et al., 2016) and depends on stimulus modality (Noel et al., 2015) and complexity (Stevenson et al., 2014). Interestingly, individuals with schizophrenia (Stevenson et al., 2017) and autism spectrum disorders (Foss-Feig et al., 2010; Stevenson et al., 2014) have been shown to have significantly wider TBWs than controls, leading to impairments in speech perception (Stevenson et al., 2018).

Using feedback, Powers et al. (2009) demonstrated a significant reduction in TBW size after a single training phase, with a mean reduction of nearly 80 ms. Whether these training effects are brought about by an improvement in temporal processing in the auditory (De Niear et al., 2016) or the visual modality (Powers et al., 2009), or both, is still an open question. Furthermore, the degree to which audiovisual simultaneity training is task and stimulus specific is still unclear. TBW training using the SJ task does not transfer to improvements in a comparable perceptual task with identical stimuli (Matthews et al., 2016) and does not reduce susceptibility to the sound-induced flash illusion, an alternative measure of audiovisual temporal acuity (Powers et al., 2016). However, training at a temporal order judgement task, with unimodal stimuli, has been shown to cause reductions in bimodal TBW size measured using the SJ task (Stevenson et al., 2013).

A hallmark of perceptual learning is stimulus specificity. The aim of our research was to investigate whether training-induced reductions in TBW size transfer across stimulus intensities. Our hypothesis was that training will reduce the TBW size, but this learning effect will not transfer across intensities.

To test our hypothesis, participants were trained on either a low or high visual intensity stimulus paired with an auditory stimulus of fixed intensity. Participants were tested with both stimulus intensities before and after training. Our main finding is that TBW size is reduced with training, but this training effect is specific to high visual intensity stimuli.

## Method

### Participants

A total of 32 participants (23 female), aged 18–28 (mean = 21.03, *SD* = 2.44), were recruited via opportunity sampling. All reported normal or corrected to normal vison and normal hearing. Two participants were removed from the final analysis as their relative frequency of *simultaneous* responses did not exceed 0.1 at any SOA in either the pretraining or posttraining dim visual stimuli condition. Participants had to achieve the following minimum performance requirements after training to be included in the analysis: (a) an overall TBW of below 1,000 ms and (b) a peak in the fitted curve between –300 and 300 ms, the range of tested values. Of the remaining participants, 11 completed the bright training condition, and 10 were trained with the low-intensity stimuli.

### Design

Participants were assigned to either the bright or the dim visual stimuli training group, and performed an SJ task consisting of interleaved bright and dim visual stimuli both before and after the assigned training condition. The stimuli in the pre- and posttraining sessions were identical in both groups.

### Apparatus

Participants were seated in anechoic chamber (IAC, Winchester, UK), 113 cm from a LED and speaker that were held at roughly eye level in an adjustable clamp. A Tucker Davies RP2.1 real-time processor (TDT technologies, Alachua, FL) was used to generate the visual and auditory stimuli. A single “Xenta M-219 Notebook speaker” produced the auditory stimuli. This was located 1.62° below a 5 mm white LED that was attenuated using three neutral density filters with a fractional transmittance of 50%, 25%, and 6.25%, respectively. A custom-built button box was used to record the participant’s responses. MATLAB (ver. R2017b) was used on a PC located outside of the booth to control the Tucker Davies system and record responses.

### Threshold Estimation

Using identical apparatus to the current experiment, eight participants (age range: 22–43, mean = 27.00, *SD* = 2.39) completed a visual threshold estimation, followed by an auditory threshold estimation using a two-interval forced-choice procedure, after 15-minute dark adaption. Two interleaved staircases with the QUEST (Watson & Pelli, 1983) threshold estimation procedures were used for each modality. In the visual threshold estimation, participants were presented with two clearly audible 100-ms beeps, with an SOA of 1 second. A 100-ms visual flash occurred 250 ms after one of the two beeps (Koenig & Hofer, 2011). The auditory threshold estimation mirrored that of the visual threshold, in that an auditory beep followed one of two clearly visible flashes. In each task, participants were asked to press the left or right button, depending on whether the target stimulus followed the first or second non-target stimulus, respectively. The final threshold was estimated to be the minimum intensity at which the participant could detect the stimulus with ∼75% accuracy.

### Stimuli

The stimuli used in the main experiments consisted of a 100-ms visual flash and a 100-ms auditory beep. The visual flash was presented at two intensities (0.02 cd/m^2^ and 1.34 cd/m^2^), which were 6 dB and 24 dB above the mean estimated visual threshold (see threshold estimation). The auditory beep had a fixed frequency of 1000 Hz and was presented with a flat tone amplitude envelope at 35.54 dB, 15 dB above the mean estimated threshold. During the training sessions, auditory beeps were used to provide feedback after each response from the participant. A correct response was defined as saying *simultaneous* for an SOA of 0 ms. A correct response was followed by two 1500 Hz tones, lasting 40 ms each and separated by 100 ms. An incorrect response was signified by a single 150 ms tone at 500 Hz. The interstimulus interval of all trials was 1,500 ms, plus a random value between 0 and 2,000 ms.

### SJ Task

Bimodal trials were presented at 11 stimulus onset asynchronies (SOAs; –300, –200, –150, –100, –50, 0, 50, 100, 150, 200, and 300 ms), with positive values indicating that the visual stimulus was leading. The stimuli were randomly interleaved and were presented 20 times at both intensity levels (bright and dim) and at each SOA, both before *and* after training. These trials were split into two pretraining and two posttraining blocks, totalling 880 trials for each participant.

### Training Task

Bimodal trials were presented at either the bright or dim visual intensity, and at only 7 SOAs (–150, –100, –50, 0, 50, 100, and 150 ms). Training-induced reductions in TBW size are dependent on the trained SOAs being suitably small (De Niear et al., 2016), and previous research has shown that a single training session at these offsets was enough to reduce TBW size (Powers et al., 2009). There were 600 trials presented in a random order across two training blocks. Within each block of the training phase, the ratio of simultaneous to non-simultaneous trials was 1:1, where the non-simultaneous trials were split evenly across the six non-simultaneous SOAs. The 1:1 ratio aimed to reduce the likelihood that the training would instil a bias, causing participants to simply reduce their overall *simultaneous* response rate and inadvertently reducing their estimated TBW size (Powers et al., 2009). Feedback was given after every response by the participant (see Stimuli subsection). The auditory feedback was presented after every response, within 150 ms of this response. No feedback was given if participants failed to respond within 2,750 ms of a stimulus offset.

### Data Analysis

The data were fitted using Yarrow et al.’s (2011) procedure. The bell-shaped simultaneity data were fitted using the difference of two cumulative Gaussians, allowing for an asymmetrical fit for the visual-leading (VA) and audio-leading (AV) responses. For each side (AV, VA), two parameters are fitted, the mean and the standard deviation of the cumulative Gaussian. Following Yarrow et al. (2011), the standard deviation is used as a measure of sensory noise (plus any variance in criterion placement). The TBW is defined as the difference between the two means (criteria) of the Gaussian curves.

We used 2 × 2 analyses of variance (ANOVAs) to assess the effect of intensity (bright or dim) and training (pre- or posttraining) on TBW size (ms). In addition, 2 × 2 × 2 ANOVAs were used to assess the effect of intensity, training, and leading modality (AV or VA) on the SDs of the fitted Gaussians. Additional post hoc comparisons were carried out using Wilcoxon Signed-rank tests.

### Procedure

The experiment began with two practice blocks consisting of 36 bright trials, split across three SOAs (–200 ms, 0 ms, and 200 ms), in which participants were instructed to press the top button if they believed that the two stimuli occurred simultaneously and the bottom button if they occurred non-simultaneously. Subsequently, participants were given a 15-minute dark adaption period, followed by two SJ blocks and a single training block. Participants were then given a 15-minute break, during which they could leave the anechoic chamber. This break aimed to reduce the impact of boredom/fatigue on posttraining performance. Participants then completed an additional 15-minute dark adaption, followed by the second training block, and two posttraining SJ blocks.

## Results

**Figure 1.**
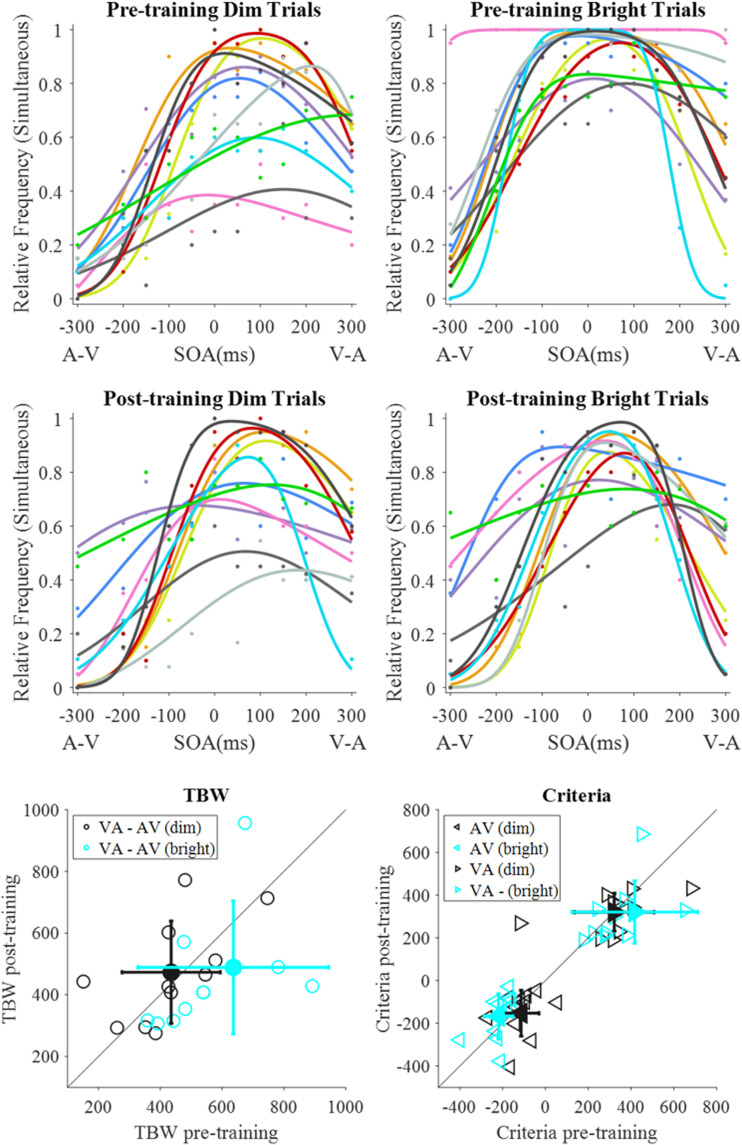
Bright training group data (*n* = 11). Rows 1 and 2 contain fits for the pretraining (row 1) and posttraining (row 2) data for the dim (left column) and bright (right column) stimuli. Each colour represents an individual observer. Row 3 consists of scatterplots of individual data, alongside means (filled-in markers) and standard deviations (error bars). Row 3 (left) represents pre- and posttraining TBW sizes, calculated as the difference between the two criteria (VA – AV), where data points below the reference line indicate a smaller TBW after training. Row 3 (right) represents the placement of the AV and VA criteria, whereby values above the reference line for the AV criterion and below the line for the VA criterion indicate a shift towards physical simultaneity following training. One data point is not shown in both scatterplots (row 3). AV = audio-leading; SOA = stimulus offset asynchrony; VA = visual-leading; TBW = temporal binding window.

**Figure 2.**
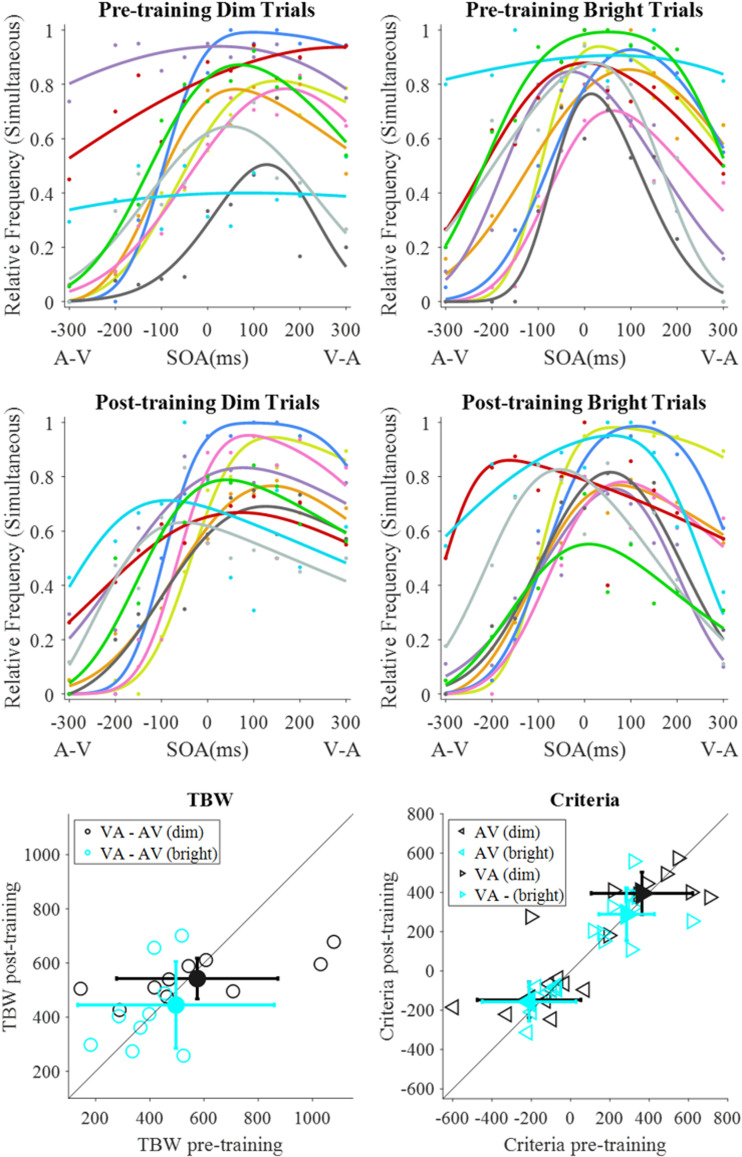
Dim training group (*n* = 10). Rows 1 and 2 consist of the individual observer fits. Row 3 contains scatterplots of the pre- and posttraining TBW size (left; one data point is not shown) and criteria placement (right). AV = audio-leading; SOA = stimulus offset asynchrony; VA = visual-leading; TBW = temporal binding window.

The primary purpose of this study was to establish whether any training-induced reductions in TBW size transfer across stimulus intensities. To test this hypothesis, we measured the TBW before and after training with either a bright (high-intensity) visual stimulus (Figure 1) or a dim (low-intensity) visual stimulus (Figure 2), and a constant auditory intensity. Both groups were then tested with dim and bright stimuli.

Each individual observer’s data were fitted with a difference of two cumulative Gaussians (Yarrow et al., 2011). The relative frequency of *simultaneous* responses is plotted as a function of SOAs, with AV on the left and VA on the right side. The first row shows the data and best fitting curves pretraining and the second row for posttraining. There is substantial variability between observers which is also reflected in the estimated TBW and the criteria (row 3). If there is no effect of training, the TBWs and criteria should be clustered around the 45-deg line; if training takes place, the TBW data points should lie below this line. For the criteria, a reduction in TBW size should be reflected in the AV criteria to lie above the 45 deg and the VA to lie below this line, indicating a shift towards the midpoint of the TBW.

For the training with bright stimuli (Figure 1), the TBW for the bright stimuli lie below this line, whereas no training effect is observed for the dim stimuli, as confirmed by the ANOVA which shows a significant interaction between pre- versus posttraining and stimulus intensity, *F*(1, 10) = 7.38, *p* = .022, *ηp*^2^ = .43, but no significant main effect of training, *F*(1, 10) = 1.21, *p* = .298, *ηp*^2^ = .11, or of stimulus intensity, *F*(1, 10) = 3.16, *p* = .106, *ηp*^2^ = .24. Post hoc tests revealed that the training with the bright stimuli led to a significant reduction in TBW size for bright stimuli, *Z* = 1.96, *p* = .025 (one-tailed), but not for dim stimuli, *Z* = 0.09, *p* = .465 (one-tailed). We then tested whether the reduction in TBW size was driven by a criterion shift on the AV, the VA, or on both sides (Figure 1, row 3). We found a significant shift towards physical simultaneity (SOA = 0) only for the AV criterion, *Z* = 1.69, *p* = .046 (one-tailed), but not the VA criterion, *Z* = 1.07, *p* = .143 (one-tailed). For the group trained with dim stimuli (Figure 2), we find no significant effect of training or stimulus intensity on the TBW size—training: *F*(1, 9) = 0.37, *p* = .559, *ηp*^2^ = .04, stimulus intensity: *F*(1, 9) = 0.56, *p* = .474, *ηp*^2^ = .06—and no significant interaction—*F*(1, 9) = 0.03, *p* = .861, *ηp*^2^ = .00.

**Figure 3.**
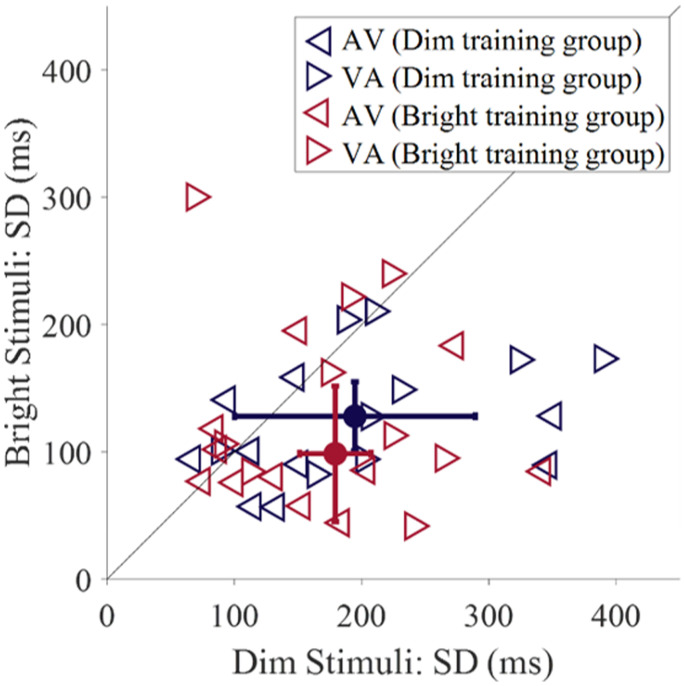
The SDs of the fitted Gaussians for audio-leading (AV) and visual-leading (VA) pretraining data, across all observers (*n* = 21). Filled-in circles and error bars represent the median and standard errors, taken across both the AV and VA fits, for the dim (purple) and bright (maroon) training groups. Three data points (> 450) are not shown. AV = audio-leading; VA = visual-leading.

Our main finding is that training reduces the TBW, but only if the observers were trained with bright stimuli (Figure 1). When observers were trained with dim stimuli, no effect of training was observed, neither for dim nor for bright test stimuli (Figure 2). This lack of learning could be due to the poor discrimination sensitivity of the SOAs for the dim stimuli as shown in Figure 3: The SD for dim stimuli is about twice as large than for bright stimuli. A 2 × 2 × 2 mixed ANOVA, investigating the effect of visual stimulus intensity (dim or bright), leading modality (AV or VA), and training group (training with dim or with bright stimuli) on the pretraining SD estimates showed that the main effect of stimulus intensity was approaching significance, with higher SDs for dim stimuli across the two training groups (Table 1). Furthermore, there was a significant main effect of leading modality, with higher SDs for VA stimuli. There was no main effect of training group, and importantly, no interaction between training group and leading modality or intensity.

**Table 1.** Summary Table for the Mixed ANOVA of Pretraining SDs.

	Predictor	Sum of squares	*df*	*F*	*p*	*η_p_* ^2^
Between-subjects effects	Training group (dim or bright)	44396	1, 19	0.35	.562	.02
Within-subjects effects	Stimulus intensity	165232	1, 19	4.10	.057*	.18
	Leading modality (A or V)	238600	1, 19	6.85	.017**	.27
	Intensity × Training Group	72354	1, 19	1.80	.196	.09
	Leading Modality × Training Group	0.51	1, 19	<0.001	.997	<.001
	Intensity × Leading Modality	25094	1, 19	0.38	.545	.02
	Intensity × Leading Modality× Training Group	92216	1, 19	1.40	.252	.07

*Note*. * and ** signify *p* values significant at the .1 and .05 levels, respectively.

Finally, we investigated the effect of training, intensity, and leading modality on the SDs of the fitted Gaussians. For the bright training group, there was a significant main effect of leading modality, *F*(1, 10) = 12.39, *p* = .006, *ηp*^2^ = .55. Participants had higher SDs to the VA stimuli than AV stimuli. The main effects of training and intensity, plus all interactions, were non-significant (*p* > .1). As for the dim training group, there was also a main effect of leading modality, *F*(1, 9) = 20.61, *p* = .001, *ηp*^2^ = .70, with higher SDs for VA stimuli. Furthermore, there was a significant main effect of intensity, *F*(1, 9) = 5.69, *p* = .041, *ηp*^2^ = .39. Observers had higher SDs for dim stimuli than for bright stimuli. All other effects were non-significant (*p* > .1).

## Discussion

Previous research has shown that the size of the TBW in audiovisual SJs can be reduced through training. Here, we tested whether training transferred across different visual signal intensities. While there was no effect of training on perceptual sensitivity (inverse of the SDs of the psychometric functions), individuals trained with bright visual stimuli showed reduced TBW size for bright stimuli after training, an effect that was driven by a criterion shift towards physical simultaneity for audio-leading bimodal stimuli, in line with De Niear et al. (2016). Importantly, this improvement did not transfer to dim stimuli. The individuals who were trained with dim stimuli showed no change in TBW, neither for dim nor for bright test stimuli. Our main experimental hypothesis that training is intensity-specific was therefore only partly supported.

The reduction in the TBW when training was performed with bright stimuli is consistent with previous reports (De Niear et al., 2016; Powers et al., 2009, 2012; Zerr et al., 2019). Our analysis also shows that this training effect is consistent with a shift in AV criterion placement, supporting evidence that trial-by-trial feedback can induce criterion shifts (Aberg & Herzog, 2012) which can lead to performance improvements in perceptual tasks (Herzog et al., 2006). The stimulus specificity of this criterion shift is consistent with perceptual learning as a mechanism underlying the performance improvement (Herzog et al., 2006).

Individuals trained with the dim visual stimuli showed *no* significant change in TBW size with training. Our data show that participants in both training groups had lower initial (pretraining) sensitivity (for dim stimuli than for bright stimuli). The reliability of stimulus-feedback combinations is an important aspect in training-induced reductions in audiovisual TBW size (De Niear et al., 2017) and in subjective confidence in perceptual judgements (Boldt et al., 2017). We speculate that a relative unreliability in the perception of audiovisual offsets for low-intensity stimuli may have impeded the effectiveness of the dim intensity training; if participants are less likely to consistently perceive simultaneity, or non-simultaneity, at a given SOA, then more perceptual variability is introduced when training these individuals to provide a specific response at said offset. In addition, consistent with previous research, observers had much lower SDs for AV in contrast to VA stimuli (Cecere et al., 2016; van Eijk et al., 2008). It has been argued that this is due to the low-level attentional effects of auditory signals, which alert the visual system to an upcoming visual stimulus (Thorne & Debener, 2014), resulting in higher sensitivities for audio-leading offsets (Cecere et al., 2016).

The ability to accurately integrate signals from different sensory modalities has potential wide-ranging effects on daily living. Increases in TBW size have been associated with increased fall risk in older age (Setti et al., 2011) and poorer performance on speech perception tasks (Conrey & Pisoni, 2006; Stevenson et al., 2018). Training healthy individuals at an audiovisual SJ task led to lasting improvements in speech perception (Zerr et al., 2019). Training with the specific aim to reduce the TBW has consequently been proposed as an intervention for those with multisensory deficiencies (Foss-Feig et al., 2010; Powers et al., 2009; Setti et al., 2014; Stevenson et al., 2017; Wallace & Stevenson, 2014). Our results highlight that, for judgements of perceived simultaneity, perceptual learning is specific for high visual intensity stimuli. Such a finding potentially highlights limitations in proposed interventions; if training is specific to a visual intensity, or intensities, then training-induced improvements may not be applicable in dynamic, real-world settings. The specificity of perceptual learning increases with training (Jeter et al., 2010), and, to be useful across a range of conditions, a careful balance between specificity and generalisation has to be struck. This consideration may also explain conflicting results, such as the generalisation of unimodal TOJ training to bimodal stimuli (e.g., Stevenson et al., 2013 vs. Zerr et al., 2019).

Previous research shows that perceptual learning is possible at near-threshold stimuli (e.g., Andersen et al., 2010; Cong et al., 2016). While our results could potentially highlight a task specific limitation, where the SJ task is untrainable at low visual intensities, we argue that a more parsimonious explanation is that in our paradigm, training is non-transferable from a high visual intensity to a low visual intensity. However, we should not discount the former, and so further research should be conducted with various visual intensities.

In conclusion, the temporal window within which audiovisual stimuli are perceived as simultaneous can be narrowed by training, but the effect of training does not transfer from the high to low visual intensity stimuli. This improvement is driven by a criterion shift for audio-leading bimodal stimuli towards physical simultaneity.
